# Designing Polymeric Multifunctional Nanogels for Photothermal Inactivation: Exploiting Conjugate Polymers and Thermoresponsive Platforms

**DOI:** 10.3390/pharmaceutics17070827

**Published:** 2025-06-25

**Authors:** Ignacio Velzi, Edith Ines Yslas, Maria Molina

**Affiliations:** 1 Departamento de Química, Universidad Nacional de Río Cuarto, Río Cuarto 5800, Argentina; ivelzi@exa.unrc.edu.ar; 2 Instituto de Investigaciones en Tecnologías Energéticas y Materiales Avanzados (IITEMA), Consejo Nacional de Investigaciones Científicas y Técnicas (CONICET), Ruta Nac 36 km 601, Río Cuarto 5800, Argentina; 3 Departamento de Biología Molecular, Universidad Nacional de Río Cuarto, Río Cuarto 5800, Argentina

**Keywords:** nanogels, polypyrrole, antimicrobial materials, photothermal inactivation, combined therapy

## Abstract

**Background/Objectives**: Photothermal therapy (PTT) is an emerging minimally invasive strategy in biomedicine that converts near-infrared (NIR) light into localized heat for the targeted inactivation of pathogens and tumor cells. **Methods and Results**: In this study, we report the synthesis and characterization of thermoresponsive nanogels composed of poly (*N*-isopropylacrylamide-*co*-*N*-isopropylmethylacrylamide) (PNIPAM-*co*-PNIPMAM) semi-interpenetrated with polypyrrole (PPy), yielding monodisperse particles of 377 nm diameter. Spectroscopic analyses—including ^1^H-NMR, FTIR, and UV-Vis—confirmed successful copolymer formation and PPy incorporation, while TEM images revealed uniform spherical morphology. Differential scanning calorimetry established a volumetric phase transition temperature of 38.4 °C, and photothermal assays demonstrated a ΔT ≈ 10 °C upon 10 min of 850 nm NIR irradiation. In vitro antimicrobial activity tests against *Pseudomonas aeruginosa* (ATCC 15692) showed a dose-time-dependent reduction in bacterial viability, with up to 4 log CFU/mL. Additionally, gentamicin-loaded nanogels achieved 38.7% encapsulation efficiency and exhibited stimulus-responsive drug release exceeding 75% under NIR irradiation. **Conclusions**: Combined photothermal and antibiotic therapy yielded augmented bacterial killing, underscoring the potential of PPy-interpenetrated nanogels as smart, dual-mode antimicrobials.

## 1. Introduction

Antimicrobial resistance (AMR) poses a significant global health threat [1,2,3], rendering conventional antibiotics increasingly ineffective and leading to a resurgence of infectious diseases that were once easily treatable [4]. The escalating prevalence of multidrug-resistant pathogens highlights the urgent need for innovative therapeutic strategies to combat this growing crisis [5,6,7]. Among the emerging technologies, photothermal therapy (PTT) has garnered significant attention as a promising approach for inactivating microbial pathogens, circumventing the shortcomings associated with traditional antimicrobial agents [8,9].

The rise of AMR is attributed to the overuse and misuse of antibiotics, which has led to the selective pressure favoring the survival and proliferation of resistant microbial strains [10,11,12]. This phenomenon is further exacerbated by the limited availability of novel antibiotics and the slow pace of drug development, resulting in a critical gap in the ability to combat resistant pathogens [13]. Consequently, infections caused by multidrug-resistant bacteria, fungi, and other microorganisms have become increasingly challenging to treat, posing a significant threat to public health and healthcare systems worldwide.

*Pseudomonas aeruginosa* is a Gram-negative bacterium known for its versatility and resilience in various environments [14,15]. It is an opportunistic pathogen, particularly dangerous to individuals with compromised immune systems, such as those with cystic fibrosis, burn wounds, or those undergoing chemotherapy. *P. aeruginosa* is notorious for its intrinsic resistance to many antibiotics and its ability to acquire additional resistance mechanisms, making infections difficult to treat. This pathogen is a significant concern in healthcare settings, where it can cause severe infections like pneumonia, urinary tract infections, and bloodstream infections [15].

In this context, PTT has emerged as a promising alternative therapeutic modality for the treatment of infections, offering several advantages over traditional antimicrobial agents [9]. By leveraging light-absorbing agents to generate localized heat and induce thermal damage to microbial cells, PTT provides a targeted and minimally invasive approach to microbial inactivation. Moreover, the non-specific mechanism of action of PTT makes it less susceptible to the development of resistance compared to conventional antibiotics, offering a viable strategy to overcome the challenges posed by AMR.

In recent years, the development of advanced materials for biomedical applications has been significantly influenced by the emergence of nanotechnology [16,17,18,19]. Among these, polymeric nanogels have garnered substantial attention due to their unique properties. These nanomaterials represent a versatile platform for the development of photothermal agents, offering inherent advantages such as high loading capacity, tunable size, and stimuli-responsive behavior [20]. Within the broad category of responsive polymers, thermoresponsive polymers are of particular interest [21] for bio applications such as cancer therapy [22] and regenerative medicine [23]. The integration of conjugate polymers and thermoresponsive elements into polymeric nanogels further enhances their photothermal properties and enables precise control over drug release kinetics, thus augmenting their therapeutic efficacy. Moreover, the biocompatibility and biodegradability of polymeric nanogels make them suitable for various biomedical applications, including targeted drug delivery and image-guided therapy [24,25]. Conjugate polymers, characterized by their π-conjugated backbone, offer unique optical and electronic properties, including high absorption coefficients and efficient photothermal conversion under near-infrared (NIR) light irradiation. Thus, conjugate polymers serve as efficient light absorbers, converting NIR light into heat and amplifying the photothermal effect within the nanogel matrix [25]. This enhanced photothermal conversion efficiency facilitates rapid and localized heating of the target site, leading to efficient destruction of pathogens or tumor cells [26,27,28].

In this work, an all-polymeric multifunctional nanogel based on poly(*N*-isopropylacrilamide-*co*-*N*-isopropylmethylacryalamide) and semi-interpenetrated with polypyrrole (NG-PPy) was developed for photothermal inactivation of microbial pathogens. The developed nanogels are considered multifunctional systems because they integrate the capacity to act as carriers for therapeutic agents, induce PTT, and modulate the release of gentamicin in a controlled manner into a single platform. What distinguishes this copolymer structure is the possibility to fine-tune the VPTT to a physiologically relevant range. By elucidating the underlying principles governing the design of these multifunctional systems and highlighting their therapeutic potential in combating multidrug-resistant infections, we seek to contribute to the ongoing efforts to develop effective strategies for the management of AMR.

## 2. Materials and Methods

### 2.1. Materials

For the synthesis of the polymeric hydrogels, the following reagents were employed: the vinyl monomers *N*-isopropylacrylamide (NIPAm, Sp2, ≥99%) and *N*-isopropylmethacrylamide (NIPMAm, Sp2, ≥99%); *N*,*N*’-methylenebisacrylamide (MBA, Sigma-Aldrich, St. Louis, MO, USA, 99%) as the cross-linking agent; potassium persulfate (KPS, Sigma-Aldrich, ≥99%) as the radical initiator; and sodium dodecyl sulfate (SDS, Riedel-de-Haën, Munich, Germany, ≥97%) as the stabilizer. For the preparation of semi-interpenetrating nanogels, pyrrole (Fluka, Buchs, Switzerland, 97%) was distilled under reduced pressure prior to use. Milli-Q water was used as the solvent in all synthesis procedures.

### 2.2. Synthesis

#### 2.2.1. Nanogel Synthesis

Nanogels were synthesized via free radical polymerization of vinyl monomers, following the procedure described by Theune et al. (2019) [29]. In a typical synthesis, NIPAm and NIPMAm were weighed in a 1:1 molar ratio to a final concentration of 123 mM. SDS was added at 1 mM, and MBA at 4.3 mM. Milli-Q water was added to reach the final volume. The mixture was deoxygenated by bubbling nitrogen (N_2_) for 30 min and then sealed. The reaction flask was placed in a glycerin bath on a hot plate at 68 °C and stirred for 1 h before the addition of KPS (1.7 mM) as the initiator. Polymerization proceeded for 4 h under constant temperature and stirring.

After synthesis, the nanogel dispersion was transferred into a dialysis membrane (Carl Roth, Karlsruhe, Germany, 50 kDa) and dialyzed against distilled water for 7 days, with frequent water changes. The resulting nanogels were lyophilized to yield a white powder.

#### 2.2.2. PPy Semi-Interpenetration

Dry nanogels were swollen in 0.1 M HCl solutions containing pyrrole at concentrations of 0.01, 0.03, and 0.05 M. The nanogel concentration was maintained at 10 mg/mL. After swelling, the nanogels were stabilized in an ice bath for 30 min to ensure consistency across assays under external conditions.

Following complete swelling, in situ polymerization was initiated by rapidly adding 0.1 M KPS solution, in a volume corresponding to half that of the pyrrole-HCl solution. Polymerization was carried out for 30, 60, or 90 min. To terminate the reaction, 100 µL of 4 M NaOH was added. The resulting blue dispersion was transferred to a dialysis membrane (Carl Roth, 50 kDa) and dialyzed against distilled water for 7 days with frequent water changes.

### 2.3. Physicochemical Characterization

The thermoresponsive nanogels (NGs) and the semi-interpenetrated networks (NG-PPy) were characterized in terms of particle size by dynamic light scattering (DLS), morphology by transmission electron microscopy (TEM), and chemical structure by Fourier-transform infrared spectroscopy (FTIR), ultraviolet-visible spectroscopy (UV-Vis), and nuclear magnetic resonance (NMR). The volume phase transition temperature was determined using differential scanning calorimetry (DSC) and DLS. Detailed experimental procedures are provided in the Supplementary Information.

### 2.4. Photothermal Properties

The photothermal efficiency (η) of thermoresponsive nanogels semi-interpenetrated with polypyrrole (NG-PPy) was assessed to determine their capacity to convert near-infrared (NIR) light into heat. Specifically, the protocol describe by Liu et al. (2014) was followed and η was calculated by Equation (1) [30].

(1) $$\eta =\frac{hs\left(T_{max}-T_{surr}\right)-Q_{dis}}{I(1-10^{Abs})}$$

where *h* is the heat transfer coefficient, *s* is the surface area of the container, *T_max_* is the maximum steady temperature of the solution and the environmental temperature is *T_surr_*, *I* is the laser power and *Abs* the absorbance of the NG-PPy at 850 nm. *Q_dis_* expresses heat dissipated from the light absorbed by the solvent and container.

For the determination of photothermal efficiency, aqueous dispersions of NG-PPy were prepared in phosphate-buffered saline (PBS, pH 7.4) at concentrations of 0.1, 0.175, and 0.35 mg/mL. For each concentration, 1 mL of the sample was transferred to quartz cuvettes (1 cm path length) and irradiated with an 850 nm NIR LED laser at a power density of 90 mW/cm^2^, with a beam diameter of 5.62 mm, and an irradiation distance of 10.5 mm for 20 min. Temperature changes (Δ*T*) were monitored in real time at 60 s intervals using a Testo 868 thermal imaging camera (Testo S.A., Buenos Aires, Argentina), and images were analyzed with IRSoft software (version 4.8). PBS without nanogels was used as a negative control under the same irradiation conditions.

To further evaluate the photothermal stability under varied conditions, thermosensitive nanogels (without PPy) were tested at a concentration of 1 mg/mL, while NG-PPy nanogels synthesized with 0.03 M pyrrole and a 60 min polymerization time were tested at concentrations of 0.1, 0.35, and 0.7 mg/mL. Each sample was prepared by diluting 100 µL of nanogel solution in 900 µL of PBS. Subsequently, 200 µL aliquots were placed into individual wells of a 96-well plate and irradiated under the same conditions (850 nm, 90 mW/cm^2^ for 20 min). Temperature data were collected with the Testo 868 thermal camera and analyzed as previously described.

### 2.5. Gentamicin Encapsulation and Release

For the encapsulation of gentamicin (Gen) into NG-PPy polymeric nanogels, a stock solution of gentamicin (100 µg/mL) was prepared in PBS. In a typical procedure, 100 µL of NG-PPy at a concentration of 0.35 mg/mL was transferred to an Eppendorf tube and centrifuged for 20 min. The supernatant was carefully removed, and 1000 µL of a 5 µg/mL gentamicin solution was added. The mixture was homogenized using a vortex.

To facilitate loading, the samples were heated to 42 °C in a dry bath to induce nanogel collapse, then cooled at room temperature for 10 min to allow the nanogels to reswell. The samples were vortexed again and incubated for 16 h to ensure efficient gentamicin encapsulation.

Gentamicin loading was quantified using a spectrofluorometer (Horiba Jobin Yvon FluoroMax-4, Horiba, Kyoto, Japan) by measuring the fluorescence intensity at an excitation wavelength of 480 nm. The fluorescence values were compared to a previously established calibration curve to determine the amount of gentamicin encapsulated. The encapsulation efficiency (*EE*) and loading capacity (*LC*) were calculated using the following equations:

(2) $$EE\;(\%)\;=\left(\frac{m_{Gen.i}-m_{Gen.f}}{m_{Gen.i}}\right)*100$$

(3) $$LC\;(\%)=\left(\frac{m_{Gen.i}-m_{Gen.f}}{m_{NG}}\right)*100$$

where $m_{Gen.i}$ is the initial mass of gentamicin added, $m_{Gen.f}$ is the mass of free (non-encapsulated) gentamicin remaining in the supernatant, and $m_{NG}$ is the mass of nanogels used for encapsulation.

To evaluate the release of gentamicin, NG-PPy nanogels at a concentration of 0.35 mg/mL were loaded with gentamicin (1 mg/mL in PBS, pH 7.4). For the release assay, 1 mL of each sample was transferred into Eppendorf tubes and exposed to three experimental conditions: 4 °C (passive diffusion control), 37 °C without irradiation, and 37 °C with near-infrared (NIR) irradiation (850 nm, 90 mW/cm^2^). In the NIR treatment, samples were irradiated for 20 min, followed by centrifugation at 10,000 rpm for 10 min. The supernatant was collected, replaced with fresh PBS, and the samples were irradiated again. This cycle was repeated three times, and an additional measurement was taken 5 h after the final irradiation.

The amount of released gentamicin was quantified from the supernatant using fluorometry, with results compared to a standard calibration curve. All conditions were tested in triplicate.

### 2.6. Biological Assays

#### 2.6.1. Preparation of Bacterial Culture

For in vitro studies, the bacterial strain *Pseudomonas aeruginosa* (ATCC 15692/PAO1) was used. The inoculum was prepared by transferring a single colony from an LB agar (Luria-Bertani) plate into liquid LB medium, followed by incubation with shaking at 37 °C for 12 h. Subsequently, 100 µL of the overnight culture was transferred into 20 mL of fresh LB medium and incubated with shaking at 37 °C for an additional 3 h, until the culture reached the exponential growth phase.

#### 2.6.2. Exposure of *P. aeruginosa* to Different Concentrations of NG-PPy

From the exponential-phase culture, 20 µL was transferred into Eppendorf tubes containing 880 µL of PBS and 100 µL of NG-PPy dispersions at concentrations of 1.75 mg/mL or 3.5 mg/mL. Control samples were prepared by adding 20 µL of the culture to 980 µL of PBS without nanogels. All treatments and controls were then transferred to a 96-well plate (200 µL per well) and incubated at 37 °C for 16 h.

#### 2.6.3. Photothermal Inactivation

After 16 h of incubation, the 96-well plate was exposed to near-infrared (NIR) light for either 10 or 20 min, according to the experimental design. Following irradiation, the samples were incubated at 37 °C for an additional 5 h. The experimental groups included:

- Control (C): Bacterial culture + PBS (no irradiation).
- Light Control (C_light_): Bacterial culture + PBS + 20 min of NIR irradiation.
- Dark Controls:
  C_1.75_: Bacterial culture + NG-PPy (1.75 mg/mL) + PBS (no irradiation).
  C_3.50_: Bacterial culture + NG-PPy (3.5 mg/mL) + PBS (no irradiation).
- Photothermal Inactivation:
  T_1.75_: Bacterial culture + NG-PPy (1.75 mg/mL) + PBS + NIR irradiation.
  T_3.50_: Bacterial culture + NG-PPy (3.5 mg/mL) + PBS + NIR irradiation.

The NIR source consisted of a series of LEDs emitting at a wavelength of 850 nm, with a power output of 3 W and an irradiance of 90 mW/cm^2^.

#### 2.6.4. Cell Viability

The microdrop method is a bacterial quantification technique that offers advantages in convenience and efficiency for plating and counting procedures. It involves the use of small-volume drops distributed on an agar plate, enabling faster and more accurate colony enumeration [31].

Following exposure of *P. aeruginosa* to different concentrations of NG-PPy or NG-PPy-Gen and subsequent NIR irradiation, serial dilutions were performed for each treatment. From each dilution, three drops of 20 µL were plated onto LB agar. The plates were incubated at 37 °C for 24 h, after which the resulting colonies were counted to determine the number of Colony-Forming Units per milliliter (CFU/mL) using the following equation:

(4) $$CFU/mL=\bar{x\;}\;colony\;count\;number\times 50\times \frac{1}{dilution\;factor}$$

### 2.7. Statistical Analysis

Statistical analysis was performed using InfoStat software (version 2020l), developed by the Applied Statistics Group at the Universidad Nacional de Córdoba. Experimental results are presented as mean ± standard deviation (SD). Differences between groups were assessed using parametric analysis of variance (ANOVA), followed by Tukey’s multiple comparison test. A significance level of *p* < 0.05 was considered.

## 3. Results

### 3.1. Synthesis and Physicochemical Characterization

In the first synthetic step, monodisperse spherical nanogels (NGs) were synthesized via precipitation polymerization, employing NIPAM and NIPMAM as monomers and BIS as the crosslinker. At 30 °C, DLS revealed a hydrodynamic diameter of 295.1 nm for the thermosensitive NG. As the temperature increased, the nanogels underwent deswelling, with their size decreasing until reaching an inflection point corresponding to the collapse of the polymer network. The fully collapsed nanogels exhibited a diameter of 160.9 nm.

**Figure 1:**
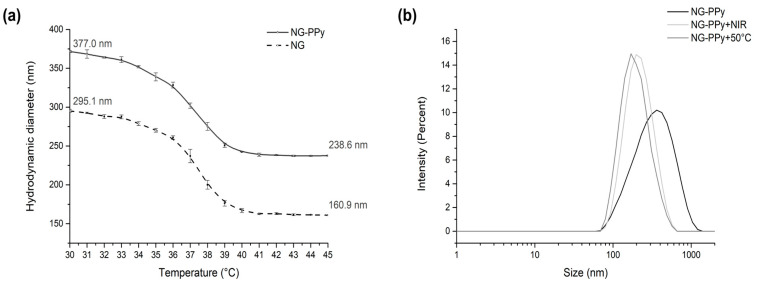
(**a**) Size (hydrodynamic diameter) vs. temperature of NG and NG-PPy. (**b**) Size of NG-PPy at 25 and 50 °C, and after 20 min of NIR irradiation.

In the second step, semi-interpenetrating nanogels (NG-PPy) were prepared by swelling the NG in a pyrrole solution, followed by polymerization initiation via rapid addition of KPS, as previously described [32]. DLS measurements at 30 °C indicated an increased diameter of 377 nm for NG-PPy, while the collapsed state yielded a size of 238.6 nm. The size expansion compared to pure NG is likely attributable to the presence of overhanging PPy chains extending from the nanogel framework, thereby increasing the overall hydrodynamic diameter. This behavior was first reported by Molina et al. (2016) in similar polymeric nanogels based on PNIPAM, hyperbranched polyglycerol, and polyaniline (PANI) [32]. In the cited work, the author showed a size increment of ~100 nm after semi-interpenetration. The temperature-dependent size variations of both NG and NG-PPy are illustrated in Figure 1.

The volume phase transition temperature (VPTT) of nanogels was determined by DSC. Both the bare nanogels (NG) and semi-interpenetrated nanogels (NG-PPy) exhibited a transition peak at 38.41 °C, confirming their comparable thermoresponsive behavior. This result aligns with previously reported values demonstrated by M. Gray et al. (2022) and others; a 50:50 monomer molar ratio was selected to achieve a VPTT near 38 °C [33]. The VPTT of the copolymer system is composition-dependent; increasing the NIPMAm molar fraction shifts the transition to higher temperatures, as reported by Urošević et al. (2018), who observed a linear increase in the lower critical solution temperature (LCST) from 32 °C to 42 °C with higher NIPMAm content [34]. These findings align with earlier work by Kokufuta et al. (2012) on the LCST behavior of PNIPAm-*co*-PNIPMAm copolymers in aqueous solutions [35]. Furthermore, Figure 1b shows the size distribution of the NG-PPy at different conditions, below (30 °C) and above (50 °C) the VPTT, and upon NIR radiation. As expected, NIR absorption induces a temperature increase, leading to the collapse of the nanogels, similarly to the effect observed upon direct thermal stimulation.

**Figure 2:**
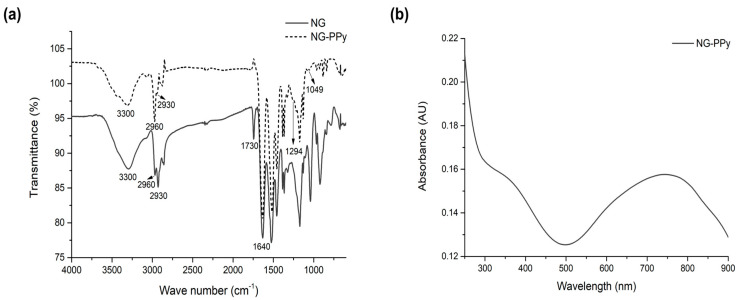
(**a**) FTIR and (**b**) UV-Vis spectroscopy of NG and NG-PPy.

To confirm the incorporation of PPy within the nanogel network, a comprehensive physicochemical characterization was conducted (Figure 2). FTIR spectroscopy of the thermosensitive NG (Figure 2a) revealed characteristic bands of the PNIPAm-*co*-PNIPMAm copolymer, including the carbonyl stretch (~1700 cm^−1^) and the N–H stretching vibration (3050–3550 cm^−1^). The FTIR spectrum of NG-PPy retained all copolymer-related bands, confirming the preservation of the polymer network. Additionally, UV-vis spectroscopy (Figure 2b) of NG-PPy exhibited two distinct PPy absorptions: a π–π * transition at 375 nm and a bipolaronic band at 750 nm, indicative of PPy in its oxidized state.

Further evidence was provided by ^1^H-NMR spectroscopy (400 MHz, D_2_O), where NG-PPy displayed signals corresponding to both PNIPAm-PNIPMAm and PPy moieties 0.84 ppm (s, 3H, NIPMAm), 1.03 ppm (s, 6H, isopropyl groups of NIPAm/NIPMAm), 1.3–2.2 ppm (m, 5H, polymer backbone protons), 3.6–4 ppm (s, 1H, NIPAm + 1H, NIPMAm), and 8.4 ppm (aromatic protons of PPy).

Altogether, the FTIR, UV-vis, and NMR data conclusively demonstrate the successful formation of semi-interpenetrating nanogels comprising a crosslinked PNIPAm-PNIPMAm network and integrated PPy.

**Figure 3:**
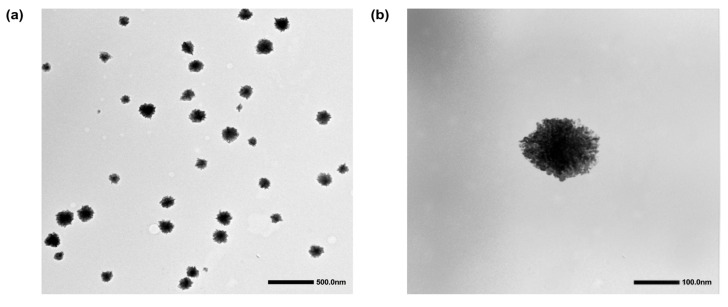
TEM images of NG-PPy at (**a**) 10k and (**b**) 80k magnification. Scale bar 500 and 100 nm, respectively.

Transmission electron microscopy (TEM) was used to further assess the morphology and shape of NG-PPy nanogels, complementing the physicochemical characterization. TEM images (Figure 3) reveal that the NG-PPy nanogels are predominantly spherical and exhibit low polydispersity. The average particle size determined by TEM (~120 nm) was notably smaller than the hydrodynamic diameter obtained by DLS, which measured 377 nm. This difference is commonly observed and can be attributed to the dehydration and shrinkage of nanogels during TEM sample preparation. Similar observations have been reported by Lou et al. (2023), who noted that the hydrodynamic diameter of Gel/HRP nanogels measured by DLS was significantly larger than the size determined via electron microscopy, attributing this to the swelling of the nanogels in aqueous media [36]. Likewise, An et al. (2023) reported that CMS6-Ly nanogels encapsulated with EGCG displayed a smaller average size by TEM compared to DLS, supporting the same interpretation [37].

In addition to their spherical shape, the TEM images reveal a distinctive contrast pattern characterized by darker central region and a lighter periphery. This may reflect a denser polymer network in the nanogel core, while the lighter outer region could correspond to semi-interpenetrated polypyrrole (PPy) chains extending outward from the core structure. The same pattern was observed previously by different groups in similar nanomaterials [32,38]. Molina et al. (2016) [32] supported this finding by the reaction procedure. At the initial stage of the reaction, the strong interaction between the aniline and PNIPAM led to a significantly higher local concentration of aniline within the nanogel (NG) interior, initiating polymerization inside the network. As the reaction progressed, polymerization primarily occurred at the surface of the nanogels. Consequently, the particles underwent a stepwise structural transformation from PNIPAM-dPG to a semi-interpenetrating network (sIPN), and ultimately formed a core–shell structure [32].

### 3.2. Photothermal Conversion

**Figure 4:**
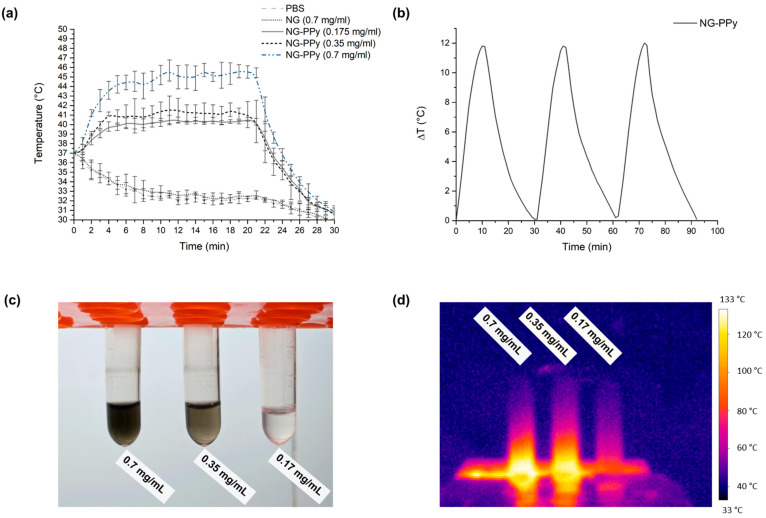
(**a**) Temperatures reached by NG-PPy at different concentrations after NIR irradiation; (**b**) photothermal stability of NG-PPy (0.35 mg/mL) over three heating cycles, showing temperature variation over time; (**c**) samples with different NG-PPy concentrations; and (**d**) comparative thermal images of NG-PPy after irradiation, captured with a thermographic camera.

The photothermal response of thermosensitive and semi-interpenetrating nanogels (sIPNs) was evaluated at various concentrations, starting from a baseline temperature of 37 °C (Figure 4a). Phosphate-buffered saline (PBS) served as the control. Upon near-infrared (NIR) irradiation, both the PBS control and thermosensitive nanogels exhibited a gradual decrease in temperature over time, similar to other non-absorbing controls. This decline can be attributed to the lack of conjugated polymers in these systems, which are essential for efficient light-to-heat conversion under NIR exposure.

In contrast, the sIPN nanogels demonstrated a marked increase in temperature over time, driven by the presence of conjugated polymers capable of absorbing NIR light and converting it into heat. The temperature rise was most pronounced between 5 and 10 min of irradiation. Among the tested concentrations, the 0.7 mg/mL sIPN nanogel suspension reached the highest temperature, followed by the 0.35 mg/mL and 0.1 mg/mL samples, which showed a concentration-dependent response. Thermographic images (Figure 4c,d) further corroborated these findings, with wells containing higher concentrations of sIPN nanogels appearing red, indicating elevated temperatures relative to the PBS control group.

The NG-PPy nanogels exhibited a photothermal conversion efficiency of 10.4% under near-infrared (NIR) irradiation, and an excellent photothermal stability upon cycles (Figure 4b) demonstrating their capacity to effectively convert light into heat. This result underscores the role of polypyrrole (PPy) as an efficient photothermal transducing polymer within the semi-interpenetrated nanogel structure. The data confirm that the incorporation of PPy not only enhances NIR absorption but also enables a significant photothermal response, positioning these nanogels as promising candidates for photothermal therapy (PTT) applications. The combination of favorable optical absorption and efficient heat generation supports their potential use in treatments where controlled hyperthermia is essential.

In comparison, Ma et al. (2022) developed h-BN/PPy composites with high light-harvesting and thermal conductivity, achieving a PCE of 82.84% at a low concentration (0.05% *w*/*w*) [39]. These materials were designed specifically for enhanced thermal conduction using boron nitride as a heat transfer enhancer and did not incorporate drug delivery or thermoresponsive hydrogel behavior. Zhang et al. (2021) reported a multifunctional yolk–shell nanoplatform (PANI/PPy@Au@MnO_2_, or PPAuMns) for cancer theranostics [40]. This complex system achieved a PCE of 59.1%, leveraging the synergistic effect of gold nanoparticles and MnO_2_ for imaging, enzymatic reactivity, and photothermal performance. However, this structure involves multiple synthetic steps and a more intricate architecture than the developed nanogels.

### 3.3. Biological Evaluation

**Figure 5:**
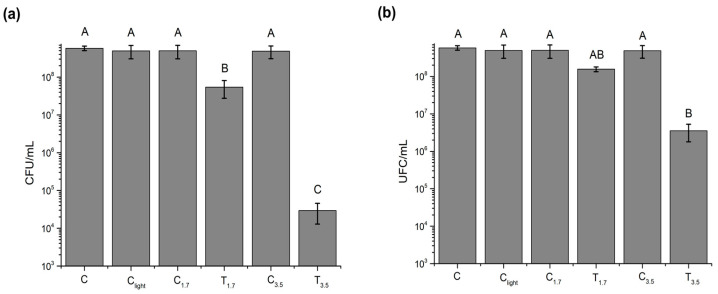
Antimicrobial effect of NG-PPy nanogels on *P. aeruginosa* viability (CFU/mL) following exposure to different NG-PPy concentrations. Bacterial viability was assessed 5 h after NIR exposure, after (**a**) 10 min and (**b**) 20 min of irradiation. Data are presented as mean ± SD, and bars with different letters indicate significant differences between treatments (*p* < 0.05).

The antimicrobial efficacy of NG-PPy nanogels was evaluated by assessing the viability of *P. aeruginosa* in the presence of varying concentrations of NG-PPy and under near-infrared (NIR) irradiation for 10 or 20 min. Bacterial viability was quantified as colony-forming units per milliliter (CFU/mL) (Figure 5). As shown in Figure 5, no significant differences were observed among the various control groups, indicating that the semi-interpenetrating nanogels alone do not exert a bactericidal effect on the Gram-negative species *P. aeruginosa* in the absence of NIR irradiation.

The antimicrobial effect of NG-PPy nanogels was further evaluated under near-infrared (NIR) irradiation at concentrations of 0.175 and 0.35 mg/mL. After 10 min of irradiation, a concentration-dependent decrease in *P. aeruginosa* viability was observed. Both concentrations exhibited statistically significant reductions in CFU/mL compared to control groups. Notably, the 0.35 mg/mL group showed a 2 log reduction in bacterial viability.

After 20 min of irradiation, this trend was further accentuated. A 1 log reduction was observed at 0.175 mg/mL, while the 0.35 mg/mL group achieved a 4 log reduction relative to the control groups. These findings highlight the dual importance of nanogel concentration and irradiation time in enhancing the effectiveness of PTT against *P. aeruginosa*.

Comparable results have been reported in other photothermal strategies. For instance, Chen et al. (2023) developed CaSiO_3_-ClO_2_@PDA-ICG (CCPI) nanoparticles for combined photothermal and photodynamic therapy against *Staphylococcus aureus* [41]. Using a laser intensity of 1 W/cm2 and nanoparticle concentration of 1 mg/mL, temperatures between 50 and 60 °C were reached, leading to a 96.7% reduction in bacterial viability after 5 min of NIR irradiation.

Overall, these findings emphasize that both the concentration of NG-PPy and the NIR exposure time are critical parameters influencing the photothermal antibacterial effect, offering a promising strategy for controlling Gram-negative pathogens like *P. aeruginosa*.

**Figure 6:**
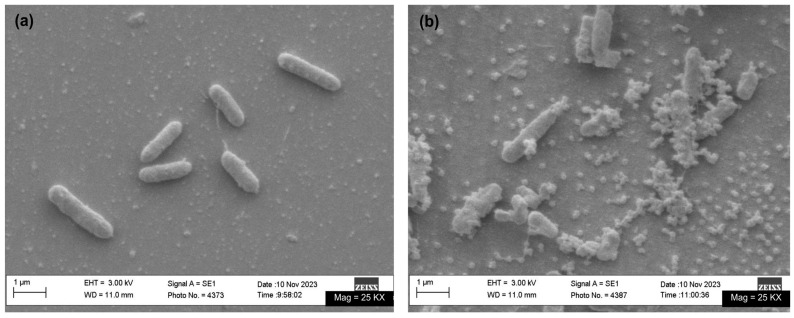
SEM images of *P. aeruginosa* (**a**) before and (**b**) after photothermal therapy with 0.7 mg/mL of NG-PPy and 20 min of irradiation.

To assess structural damage, SEM images of *P. aeruginosa* were captured before and after photothermal inactivation. In Figure 6a, the bacteria exhibit their characteristic rod-shaped morphology with intact cell membranes. In contrast, Figure 6b shows significant changes following photothermal treatment, where NG-PPy nanoparticles are visibly aggregated on the bacterial surface, suggesting interaction with the cell membrane and potential disruption due to localized heating. This arrangement suggests a specific interaction between NG-PPy and the bacterial membrane, which could promote localized heat accumulation and enhance the destructive effect of NIR irradiation. The precise localization of these nanoparticles is crucial, as it maximizes damage at the target site while minimizing impact on the surrounding cells.

Detailed SEM analysis reveals marked differences between the control group of *P. aeruginosa* and those treated with NG-PPy under NIR light irradiation. In the control group, bacterial cells maintain their typical rod-shaped morphology with rounded ends and a smooth, continuous surface, indicating that the experimental conditions alone did not cause visible structural damage.

In contrast, *P. aeruginosa* cells treated with NG-PPy and subjected to NIR irradiation exhibit pronounced morphological alterations. Many cells appear truncated at the ends, suggesting compromised structural integrity. Additionally, a noticeable reduction in cell size is observed, which may reflect cellular contraction or leakage of intracellular contents as a result of damage.

The surface of the treated cells also presents increased heterogeneity, with regions of roughness and irregular texture replacing the smooth membranes seen in the control. These changes point toward disruption of the bacterial cell envelope, likely due to localized photothermal effects generated by NG-PPy nanoparticles under NIR exposure.

To develop a combined therapeutic system, gentamicin (Gen) was encapsulated into NG-PPy nanogels. The loading efficiency was determined via fluorescence measurements by comparing the fluorescence intensity of the solution to a previously established calibration curve, enabling precise quantification of the encapsulated drug. The encapsulation efficiency of Gen in NG-PPy nanogels was calculated to be 38.7% (Equation (2)). While this represents a moderate efficiency, it may be sufficient depending on the therapeutic requirements and dosage needs.

In contrast, the loading capacity (Equation (3)) was remarkably high, reaching 99.48%, indicating that nearly all the initially available Gen was successfully incorporated into the nanogels. This high loading capacity is advantageous, as it allows for the delivery of elevated drug concentrations in small volumes, potentially enhancing the efficacy and control of gentamicin administration in clinical applications. This high loading capacity can be attributed to π–π interactions between the aromatic rings of gentamicin and the PPy chains, which likely facilitate strong molecular association and stable encapsulation within the nanogel matrix.

**Figure 7:**
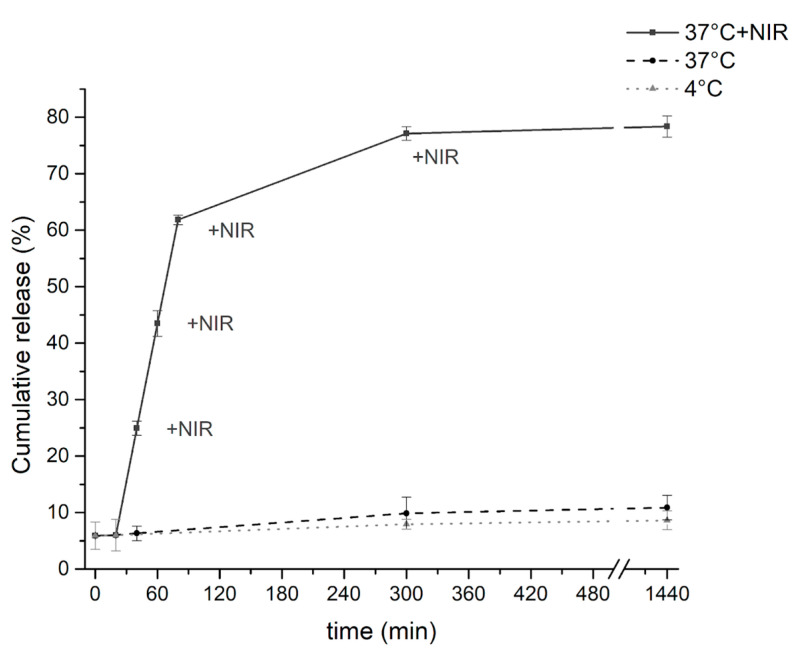
Cumulative release of Gen from NG-PPy nanogels under three different conditions: storage at 4 °C, incubation at 37 °C non-irradiated, and incubation at 37 °C with NIR irradiation (850 nm, 90 mW/cm^2^).

The cumulative release of gentamicin from NG-PPy nanogels was assessed under three different conditions: 4 °C, 37 °C without irradiation, and 37 °C with NIR irradiation. At 4 °C and 37 °C without NIR exposure, drug release remained minimal, reaching only 7–10% after 5 h (Figure 7). This indicates that, in the absence of external stimuli, the nanogels effectively retain the encapsulated drug, supporting the high loading capacity value. In contrast, under NIR irradiation at 37 °C, the cumulative release exceeded 75%, demonstrating the strong responsiveness of the system to photothermal stimulation. Remarkably, each 20 min irradiation cycle induced an additional ~20% release, supporting a stimulus-responsive, pulsed release profile. These findings underscore the potential of NG-PPy nanogels as intelligent, controlled-release platforms capable of stable drug retention under physiological conditions and efficient, localized, and repeatable release upon demand through NIR activation.

**Figure 8:**
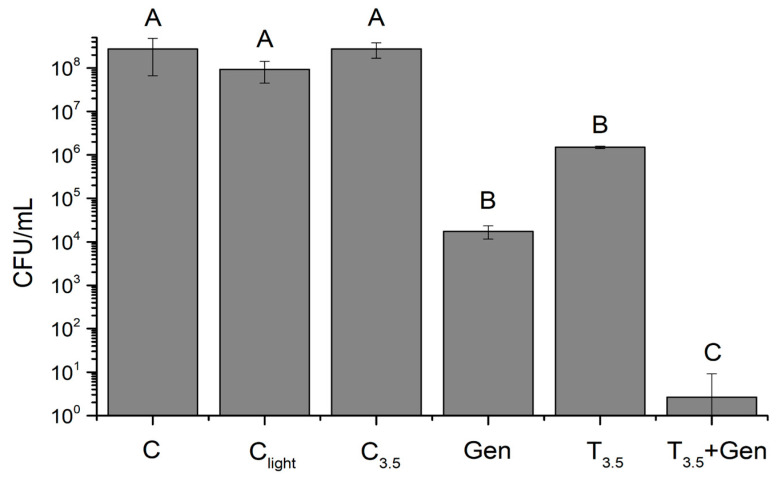
Bacterial viability (CFU/mL) of *P. aeruginosa* under different treatments: Control (C), Light Control (C light), Dark Controls (C_3.50_ NG-PPy 3.5 mg/mL), Gen, NG-PPy 3.5 mg/mL + NIR irradiation (T_3.50_) and after combined therapy with NG-PPy-Gen + NIR (T_3.50_-Gen). Values are presented as mean ± SD, and bars with different letters indicate significant differences between treatments (*p* < 0.05).

Building upon the promising results of stimulus-responsive Gen release, the antimicrobial efficacy of the combined therapy using Gen-loaded nanogels under NIR irradiation (NG-PPy-Gen + NIR) was evaluated through a microdroplet assay (Figure 8). This approach allowed for a comprehensive assessment of the therapeutic potential of NG-PPy as both a drug delivery system and a photothermal agent against *P. aeruginosa*. The minimum inhibitory concentration (MIC) and minimum bactericidal concentration (MBC) values of free Gen were determined to be 4 µg/mL and 6 µg/mL, respectively, under the same experimental conditions. Considering these values, and taking into account the high drug loading efficiency of the NG-PPy (>98%), the concentrations used in the combined photothermal–antibacterial treatments were selected to match the effective therapeutic range observed for free Gen. This allowed for a direct comparison of the antibacterial efficacy between the nanogel formulation and the free drug.

Statistically significant reductions in bacterial viability were observed in treatments involving either Gen or photothermal therapy (NG-PPy + NIR) when compared to the control groups. Notably, photothermal treatment with NG-PPy for 20 min and Gen exposure individually demonstrated considerable antibacterial activity.

Consistent with previous studies demonstrating the efficacy of antimicrobial agents and photothermal strategies, the combined NG-PPy-Gen + NIR treatment resulted in the greatest reduction in bacterial viability, significantly surpassing all control and individual treatment groups. These results further support a synergistic interaction between photothermal therapy and gentamicin delivery.

The observed synergy may be attributed to several mechanisms, including increased membrane permeability due to thermal stress, enhanced diffusion of Gen into damaged bacterial cells, and possible oxidative stress induced by NIR irradiation. Furthermore, the NG-PPy nanogels provide a dual-functional platform capable of both localized drug delivery and targeted photothermal activation. This ensures concentrated therapeutic action at the infection site while minimizing potential side effects on surrounding tissues, positioning NG-PPy-Gen + NIR as a highly promising strategy for advanced antimicrobial therapy.

## 4. Conclusions

In summary, this study successfully developed and characterized thermoresponsive semi-interpenetrating nanogels (NG-PPy) based on PNIPAm-*co*-PNIPMAm and PPy, demonstrating their potential as multifunctional platforms for advanced antimicrobial therapy. The nanogels exhibited well-defined spherical morphology, thermoresponsive behavior with a VPTT near physiological temperature, and effective photothermal conversion under NIR irradiation. Importantly, NG-PPy nanogels displayed significant antimicrobial activity against *P. aeruginosa* under photothermal conditions, with enhanced bactericidal effects observed at 0.35 mg/mL and 20 min irradiation time. The incorporation of Gen further expanded their therapeutic capacity, enabling a synergistic response when combined with NIR-triggered photothermal therapy. This dual functionality was supported by efficient drug loading, stable encapsulation under physiological conditions, and controlled, stimuli-responsive release. The combined treatment (NG-PPy-Gen + NIR) achieved the greatest reduction in bacterial viability, demonstrating superior efficacy over individual approaches. Overall, NG-PPy nanogels offer a promising strategy for targeted, on-demand antimicrobial interventions, particularly in combating resistant Gram-negative pathogens, and hold great potential for future clinical translation in localized infection management.

## Data Availability

Dataset available on request from the authors.

## Supplementary Materials

The following supporting information can be downloaded at: https://www.mdpi.com/article/10.3390/pharmaceutics17070827/s1, Details of characterization techniques.
