# Supplementary material for: Designing Polymeric Multifunctional Nanogels for Photothermal Inactivation: Exploiting Conjugate Polymers and Thermoresponsive Platforms
Source: Pharmaceutics. 2025 Jun 25;17(7):827. doi: 10.3390/pharmaceutics17070827 (PMC12300760; doi:10.3390/pharmaceutics17070827)
Supplement: Supplementary file 1 [file pharmaceutics-17-00827-s001.zip › pharmaceutics-3647668-supplementary.pdf]

Designing Polymeric Multifunctional Nanogels for Photothermal Inactivation: Exploiting Conjugate Polymers and Thermoresponsive Platforms

Ignacio Velzi [1,2,3], Edith Ines Yslas [2,3,*] and Maria Molina [1,2,*]

[1] Departamento de Química, Universidad Nacional de Río Cuarto, Río Cuarto 5800, Argentina
[2] Instituto de Investigaciones en Tecnologías Energéticas y Materiales Avanzados (IITEMA), Consejo Nacional de Investigaciones Científicas y técnicas (CONICET), Ruta Nac 36 km 601, Río Cuarto 5800, Argentina
[3] Departamento de Biología Molecular, Universidad Nacional de Río Cuarto, Río Cuarto 5800, Argentina

* Corresponding author: eyslas@exa.unrc.edu.ar (E.I.Y.); mmolina@exa.unrc.edu.ar (M.M.)

Supplementary Information

Physicochemical characterization

The hydrodynamic diameter of nanogels was measured by Dynamic Light Scattering (DLS) using an Autosizer Malvern 4700. Measurements were performed at 25 °C, 50 °C, and upon NIR irradiation, using 1 mL of a 1 mg/mL nanogel suspension in phosphate-buffered saline (PBS). The size and polydispersity index were determined from three independent measurements, with each treatment prepared in duplicate. The morphology of the nanogels was characterized by transmission electron microscopy (TEM) using a JEM 1200 Ex II microscope (JEOL, Japan). For this, 50 µL of a 10 mg/mL nanogel suspension was deposited onto copper grids coated with Formvar (250 mesh) and air-dried for 5 minutes. Visualization of the bare nanogels required staining with 1% (w/v) phosphotungstic acid. Image analysis and measurements were conducted using Digital Micrograph™ software (Gatan, Inc., Japan).

The thermal phase transition behavior of the nanogels was assessed by Differential Scanning Calorimetry (DSC), employing a heating ramp from −20 °C to 60 °C at a rate of 10 °C/min. Moreover, the degradation temperature (T_d) was studied by DSC, employing a heating ramp from 20 °C to 500 °C at a rate of 10 °C/min. To investigate the chemical structure, lyophilized samples of PNIPAm-co-PNIPMAm and NG-PPy were dissolved in deuterated water (D_2O) and analyzed using a Bruker Ascend™ TM400 NMR spectrometer operating at 400 MHz. Fourier Transform Infrared Spectroscopy (FT-IR) was also performed using a Nicolet Impact 400 spectrophotometer, recording spectra in the 600–

4000 cm^{-1} range with a resolution of 4 cm^{-1} at room temperature. Samples were prepared as KBr pellets by mixing and compressing the lyophilized nanogels with potassium bromide.

UV-Vis spectroscopy was used to evaluate the optical absorption capacity of the thermoresponsive nanogels semi-interpenetrated with polypyrrole (NG-PPy) and to confirm the incorporation of polypyrrole (PPy) into the nanogel polymeric network. This technique also facilitated verification of the semi-interpenetrated structure of NG-PPy through the analysis of its characteristic absorption bands. Aqueous solutions of NG-PPy were prepared at concentrations of 0.1, 0.175, and 0.35 mg/mL in PBS buffer (pH 7.4), and 1 mL of each was placed in 1 cm path length quartz cuvettes. PBS was used as a blank. The absorption spectra were recorded from 250 to 900 nm using a UV-Vis spectrophotometer (Hewlett–Packard, Model 8452A).
